# Soft-Tissue and Fascial Reconstruction of Vesicocutaneous Bladder Fistulas Using Pedicled Chimeric Superficial Circumflex Iliac Artery Perforator (SCIP) Flaps: A Systematic Review

**DOI:** 10.7759/cureus.103365

**Published:** 2026-02-10

**Authors:** Ibrahim Adil Hamadelniel Alhadi, Muhammad Qaiser Aziz khan, Shashwat Shetty, Jamal Khan

**Affiliations:** 1 General Surgery, The Royal College of Surgeons of Edinburgh, Edinburgh, GBR; 2 Human Anatomy/General Surgery, University of Gezira, Wad Madani, SDN; 3 Cardiac Surgery, Liaquat National Hospital and Medical College, Karachi, PAK; 4 Orthopaedics, Hillingdon Hospital, Uxbridge, GBR; 5 Internal Medicine, Pakistan Institute of Medical Sciences, Islamabad, PAK

**Keywords:** bladder fistula, chimeric flap, pelvic reconstruction, scip flap, soft-tissue reconstruction, vesicocutaneous fistula

## Abstract

Vesicocutaneous bladder fistulas are rare but debilitating, often resulting from pelvic surgery, radiotherapy, trauma, or chronic infection, and pose significant reconstructive challenges due to scarred tissue and impaired vascularity. Pedicled chimeric superficial circumflex iliac artery perforator (SCIP) flaps, incorporating both cutaneous and fascial components on a single vascular pedicle, offer thin, pliable coverage with structural reinforcement while minimizing donor-site morbidity. This systematic review, conducted according to Preferred Reporting Items for Systematic reviews and Meta-Analyses (PRISMA) 2020 guidelines, identified three case series reporting SCIP flap use for vesicocutaneous fistula reconstruction. All studies demonstrated high flap survival, successful fistula closure, and minimal donor-site complications, even in irradiated or previously operated fields. The fascial component provided additional support to bladder closure, potentially reducing recurrence, while the thin cutaneous tissue allowed adequate contouring in pelvic and infra-umbilical regions. Limitations include small sample sizes, single-center designs, and heterogeneous patient populations. Despite these constraints, pedicled and chimeric SCIP flaps appear reliable and versatile for complex vesicocutaneous bladder fistula repair. Further prospective, multicenter studies with standardized outcomes are needed to validate these findings and optimize reconstructive strategies.

## Introduction and background

Vesicocutaneous bladder fistulas are an uncommon but debilitating form of urinary fistula characterized by an abnormal epithelialized tract between the urinary bladder and the skin [1]. They most frequently arise following pelvic surgery, radiotherapy, trauma, chronic infection, or complex congenital urological conditions. Although the exact incidence is low, vesicocutaneous fistulas are increasingly encountered in tertiary referral centers due to the growing number of pelvic oncological procedures, complex reconstructive surgeries, and improved long-term survival of cancer patients [2]. These fistulas result in persistent urinary leakage, recurrent infection, skin maceration, and significant physical and psychological morbidity.

The pathophysiology of vesicocutaneous bladder fistulas is closely linked to compromised tissue vascularity, chronic inflammation, fibrosis, and repeated surgical interventions [3]. Radiotherapy and prior pelvic surgery cause microvascular damage, tissue hypoxia, and impaired collagen remodeling, rendering simple fistula excision and primary closure unreliable. Consequently, recurrence rates remain high when vascularized tissue is not interposed between the bladder and the skin. Durable reconstruction therefore requires not only closure of the fistulous tract but also restoration of healthy, well vascularized soft tissue to separate suture lines and obliterate dead space.

A variety of reconstructive techniques have been described for vesicocutaneous fistula repair. Traditional approaches include omental flap interposition, rectus abdominis muscle flaps, and gracilis muscle flaps, all of which provide robust vascularity and improve healing in compromised tissue beds [4]. However, these techniques may be associated with significant donor-site morbidity, excessive bulk, abdominal wall weakness, and functional impairment. In recent years, perforator-based flaps have gained attention due to their ability to provide thin, pliable tissue while preserving underlying muscle and reducing donor-site morbidity. Among these, the superficial circumflex iliac artery perforator (SCIP) flap offers favorable anatomy, reliable perfusion, minimal donor-site morbidity, and a concealed donor scar [5].

The pedicled chimeric SCIP flap represents a further refinement of this reconstructive approach, allowing independent positioning of cutaneous and fascial components on a single vascular pedicle. This design is particularly attractive for vesicocutaneous bladder fistula reconstruction, where both superficial skin coverage and deeper fascial reinforcement of the bladder repair are required. The inclusion of a vascularized fascial component may enhance mechanical support of the bladder closure and reduce recurrence in scarred or irradiated fields. Despite these theoretical advantages, the use of pedicled chimeric SCIP flaps for vesicocutaneous bladder fistula repair remains sparsely reported, with existing evidence limited to technical reports and small case series.

The primary aim of this systematic review is to evaluate the role and reported outcomes of pedicled chimeric SCIP flaps in the soft-tissue and fascial reconstruction of vesicocutaneous bladder fistulas. The secondary aims are to compare outcomes and complications with established reconstructive techniques, to analyze anatomical and pathophysiological factors influencing flap selection, and to identify gaps in the current literature that may guide future reconstructive strategies.

## Review

Materials and methods

Search Strategy

A systematic literature search was conducted across major biomedical databases, including PubMed/MEDLINE (Medical Literature Analysis and Retrieval System Online), Embase, Scopus, and the Cochrane Library from August 1, 2025, to December 21, 2025, to identify studies reporting vesicocutaneous bladder fistula reconstruction using pedicled chimeric or conventional SCIP flaps. The search strategy combined controlled vocabulary (Medical Subject Headings (MeSH) terms for PubMed, Emtree for Embase) and free-text keywords. Complete search strategies for each database are provided in Table 1 to ensure reproducibility. Search terms included: “vesicocutaneous fistula”, “bladder fistula”, “urinary fistula”, “SCIP flap”, “chimeric flap”, “perforator flap”, “superficial circumflex iliac artery”, and “pelvic reconstruction”, linked with Boolean operators (AND, OR) and adjacency operators where supported.

**Table 1:** Systematic search strategy for vesicocutaneous bladder fistula reconstruction using SCIP flaps SCIP: superficial circumflex iliac artery perforator

Database	Date Searched	Controlled Vocabulary/Index Terms	Free-Text Keywords	Boolean/Adjacency Operators
PubMed / MEDLINE	August 1, 2025-December 21, 2025	MeSH: "Fistula, Urinary"[Mesh], "Bladder"[Mesh], "Flaps, Surgical"[Mesh], "Perforator Flap"[Mesh]	"vesicocutaneous fistula", "bladder fistula", "urinary fistula", "SCIP flap", "chimeric flap", "superficial circumflex iliac artery", "perforator flap", "pelvic reconstruction"	AND / OR
Embase		Emtree: 'urinary fistula'/exp, 'bladder'/exp, 'surgical flap'/exp, 'perforator flap'/exp	"vesicocutaneous fistula", "bladder fistula", "urinary fistula", "SCIP flap", "chimeric flap", "superficial circumflex iliac artery", "perforator flap", "pelvic reconstruction"	AND / OR / NEAR/3
Scopus		N/A (Scopus uses keywords)	TITLE-ABS-KEY("vesicocutaneous fistula" OR "bladder fistula" OR "urinary fistula") AND TITLE-ABS-KEY("SCIP flap" OR "chimeric flap" OR "perforator flap" OR "superficial circumflex iliac artery")	AND / OR
Cochrane Library		MeSH: [Urinary Fistula], [Bladder], [Surgical Flaps]	"vesicocutaneous fistula", "bladder fistula", "urinary fistula", "SCIP flap", "chimeric flap", "perforator flap", "superficial circumflex iliac artery", "pelvic reconstruction"	AND / OR

Only studies for which sufficient methodological and outcome data were available in English were included to allow reliable data extraction without formal translation. This language restriction represents a potential source of bias, as relevant studies published in other languages may have been excluded, potentially affecting the completeness of the evidence synthesis. The reference lists of all included studies and relevant narrative reviews were manually screened (“snowballing”) to identify additional eligible data not captured by database queries. Duplicate records were removed using automated software and manual verification. Two reviewers independently screened titles, abstracts, and full-text articles using predefined eligibility criteria, with discrepancies resolved by consensus to reduce selection bias. The search methodology adhered to PRISMA 2020 standards, and the overall approach was designed to maximize sensitivity for a rare surgical condition while maintaining specificity through structured Boolean logic and controlled terminology usage [6]. No attempts were made to pool prevalence estimates or perform meta-analysis due to anticipated heterogeneity in study design, sample size, and outcome reporting.

Eligibility Criteria

Eligibility criteria were defined a priori using a PICO (Population, Intervention, Comparator, and Outcome) framework to ensure methodological consistency and clinical relevance [7]. Studies were eligible if they included adult or adolescent patients with vesicocutaneous bladder fistulas of any etiology (Population), managed using pedicled SCIP flaps, including chimeric configurations with fascial, adipofascial, or cutaneous components designed for bladder or soft-tissue reconstruction (Intervention). Studies with alternative reconstructive methods, such as rectus abdominis, gracilis, or omental flaps, were included only when used as comparators (Comparator), although comparator arms were not mandatory due to expected scarcity in this specialized field. Eligible studies were required to report at least one clinically relevant outcome, such as successful fistula closure, recurrence, urinary leak, infection, donor-site morbidity, or need for reoperation (Outcome).

Accepted study designs included prospective or retrospective cohorts and comparative observational studies. Animal studies, cadaveric anatomical studies, conference abstracts without full reporting, reviews, editorials, and expert opinions were excluded. Only studies for which sufficient methodological and outcome data were available in English were considered, and no restrictions were applied on publication year to maximize capture of relevant reconstructive data in this rare surgical domain.

Study Selection

Study selection was conducted in accordance with predefined eligibility criteria following PRISMA 2020 guidance. After removal of duplicates through automated software and manual verification, two independent reviewers screened titles and abstracts for potential eligibility, with full-text articles obtained for all records meeting initial criteria. Full texts were then assessed against inclusion and exclusion criteria using a structured screening template to ensure uniform application. Discrepancies between reviewers were resolved through discussion and consensus, and when necessary, adjudicated by a senior author to minimize selection bias. Reference lists of included studies and relevant reviews were hand-searched to identify additional eligible publications not captured during the primary search (snowballing). The selection process was documented in a PRISMA flow diagram to enhance methodological transparency and reproducibility.

Data Extraction

Data extraction was performed using a standardized data collection form developed a priori to ensure consistency and reduce transcription errors. Extracted variables included study characteristics (authors, year, country, design), patient demographics, fistula etiology, preoperative risk factors (e.g., radiation, prior surgeries), anatomical details of the defect, flap design and components, operative technique, perioperative management, follow-up duration, and clinically relevant outcomes such as fistula closure rates, recurrence, infection, donor-site morbidity, urinary leakage, and reoperation. Two reviewers independently extracted data, with discrepancies reconciled through consensus to ensure data integrity. When numerical data were incomplete, attempts were made to contact study authors for clarification; otherwise, missing data were reported as not available. Extracted data were tabulated for qualitative synthesis and comparative evaluation.

Risk of Bias Assessment

Risk of bias was evaluated independently by two reviewers using the Joanna Briggs Institute (JBI) Critical Appraisal Tools appropriate to the methodological design of each included study (e.g., cohort studies, case series) [8]. The JBI framework enabled structured assessment across domains, including clarity of inclusion criteria, completeness of clinical information, validity of outcome measures, handling of confounding factors, adequacy of follow-up duration, and appropriateness of statistical analysis where applicable. Studies were appraised using standardized checklists to ensure consistency, and scores were not used for exclusion but rather to contextualize methodological quality and interpretability of reported outcomes. The results of the risk-of-bias assessment informed the narrative synthesis and weighting of clinical evidence within the review.

Data Synthesis

Due to expected clinical and methodological heterogeneity across studies in terms of patient characteristics, fistula etiology, radiation history, flap composition, and outcome reporting, a qualitative narrative synthesis approach was adopted rather than a quantitative meta-analysis. Extracted data were grouped thematically to compare reconstructive strategies, assess closure success, evaluate complication profiles, and contextualize donor-site morbidity. When comparator techniques were present, outcomes were discussed descriptively to identify relative advantages and limitations. Given the rarity of vesicocutaneous bladder fistulas and the specialized nature of SCIP reconstruction, effect size pooling was deemed inappropriate, and findings were instead synthesized to elucidate reconstructive principles, indications, and knowledge gaps that may guide future prospective data collection and clinical research.

Protocol and Registration

This systematic review was conducted in accordance with the PRISMA 2020 guidelines to ensure methodological transparency, reproducibility, and completeness of reporting. Although the review protocol was carefully developed a priori, it was not registered in PROSPERO (International Prospective Register of Systematic Reviews) due to time constraints and the rare nature of vesicocutaneous bladder fistula studies, which represents a limitation regarding transparency and protocol availability. Nevertheless, all steps of the review, including search strategy, study selection, data extraction, and risk-of-bias assessment, were systematically performed and documented to maintain rigor and reduce bias.

Results

Study Selection Process

**Figure 1:**
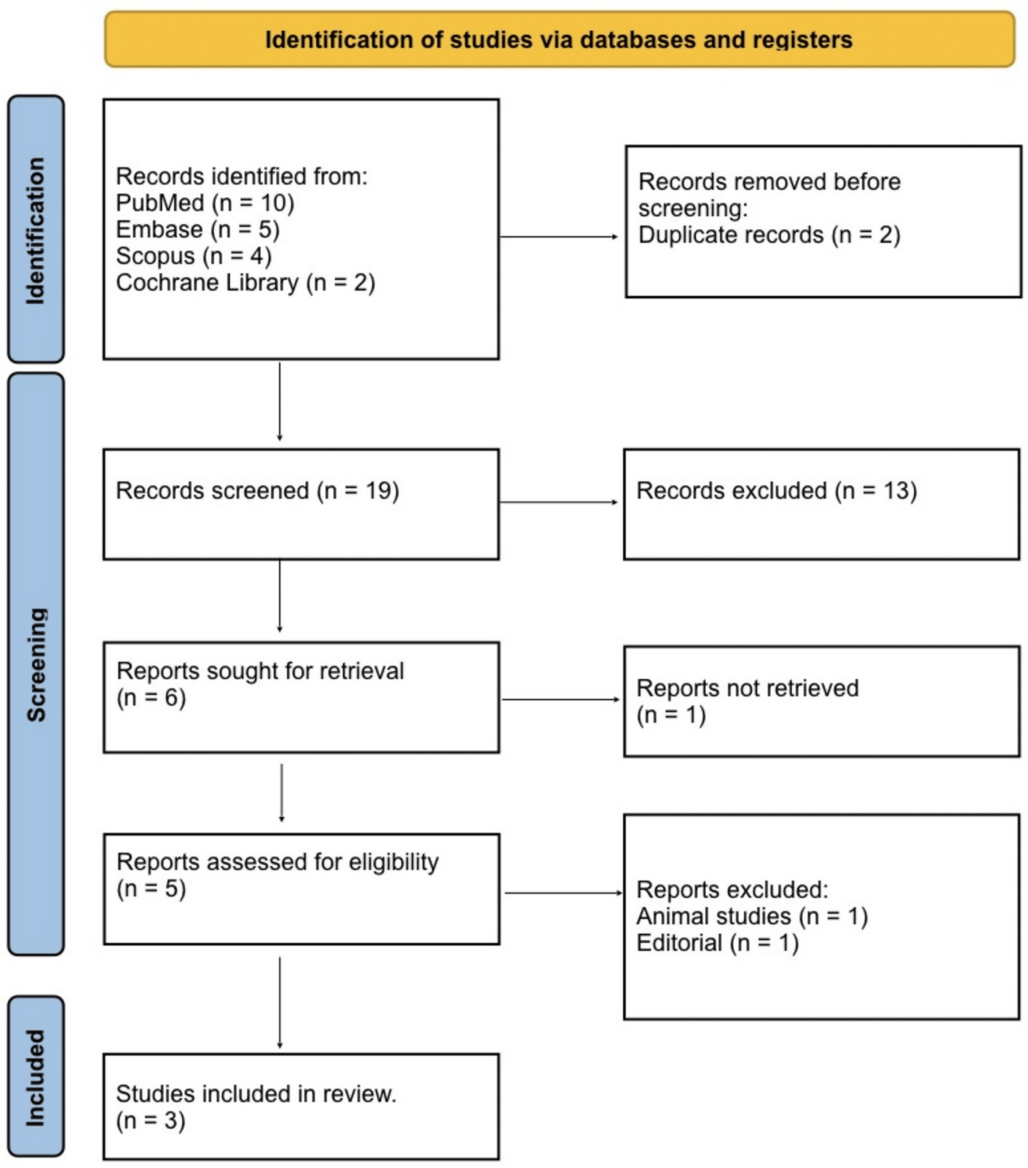
PRISMA 2020 flow diagram PRISMA: Preferred Reporting Items for Systematic reviews and Meta-Analyses

A total of records were identified through database searching, including PubMed (n = 10), Embase (n = 5), Scopus (n = 4), and the Cochrane Library (n = 2), resulting in an initial total of 21 records. After removal of duplicate records (n = 2) through automated and manual verification, 19 unique records remained for title and abstract screening. During the screening phase, 13 records were excluded due to irrelevance to vesicocutaneous bladder fistula reconstruction or SCIP flap intervention. Full-text articles were retrieved for six potentially eligible studies, of which one could not be obtained despite efforts to contact authors. Full texts were then assessed against predefined eligibility criteria, resulting in the exclusion of one editorial and one animal study. Ultimately, three studies met all inclusion criteria and were included in the systematic review. The study selection process is summarized in the PRISMA flow diagram (Figure 1), ensuring transparency and reproducibility of study inclusion and exclusion. 

Characteristics of the Selected Studies

Table 2 shows a summary of the included studies evaluating the use of pedicled chimeric and conventional SCIP flaps for soft-tissue and fascial reconstruction in vesicocutaneous bladder fistulas and related urogenital defects. Lichtenberg et al. (2024) reported on patients with complex infra-umbilical and pelvic soft-tissue defects managed using a pedicled chimeric SCIP flap, incorporating a fascial or muscle-fascial component on a single vascular pedicle [9]. The study demonstrated successful defect closure and stable soft-tissue coverage in all cases, despite underlying chronic inflammation, fibrosis, and compromised vascularity. Anatomically, the thin skin paddle provided adequate superficial coverage, while the vascularized fascial component reinforced the repair. No early complications were reported, and late outcomes showed no flap failure or wound breakdown. De Gelder et al. (2024) evaluated patients undergoing genital and urogenital reconstruction using pedicled SCIP flaps, comparing outcomes with historical techniques such as radial forearm free flaps and anterolateral thigh flaps [10]. The study highlighted high flap survival and satisfactory functional outcomes, with the thin, pliable tissue effectively reducing fistula risk and reaching the perineal and lower abdominal regions. Early complications were minor, whereas late complications included occasional urethral fistula or stricture. Sidhoum et al. (2017) focused on patients requiring thin soft-tissue reconstruction using free and pedicled SCIP flaps, comparing them with other local or perforator flaps [11]. The study reported reliable flap survival and low donor-site morbidity, facilitated by consistent perforator anatomy, which enabled flap use even in compromised tissue beds. The flaps provided good contour restoration with minimal bulk, with rare early partial congestion and minimal late donor-site morbidity. Collectively, these studies underscore the versatility, reliability, and functional advantages of SCIP-based flaps for complex pelvic and urogenital soft-tissue reconstruction, particularly in compromised or irradiated fields. 

**Table 2:** Characteristics of the selected studies SCIP: superficial circumflex iliac artery perforator flap; RFFF: radial forearm free flap; ALT: anterolateral thigh flap Pedicled flap: Flap transferred while maintaining its native vascular pedicle; Fascial component: vascularized fascia included with the flap for structural support; Infra-umbilical: area below the umbilicus (navel); Urogenital reconstruction: surgical reconstruction of urinary and/or genital structures; Donor-site morbidity: complications or functional deficits at the tissue harvest site

Authors and Year	Population (P)	Exposure/Condition (I)	Comparator (C)	Techniques	Outcomes (O)	Pathophysiological Findings	Anatomical Impact
Lichtenberg et al., 2024 [9]	Patients with complex infra-umbilical and pelvic soft-tissue defects	Pedicled chimeric SCIP flap	None (technical case series)	Harvest of SCIP flap with chimeric fascial/muscle-fascial component on single pedicle	Successful defect closure; stable soft-tissue coverage	Chronic inflammation, fibrosis, compromised vascularity	Thin skin paddle for coverage; fascial component for reinforcement
De Gelder et al., 2024 [10]	Patients undergoing genital and urogenital reconstruction	Pedicled SCIP flap	Historical techniques (RFFF, ALT)	Pedicled SCIP elevated on superficial circumflex iliac vessels	High flap survival; satisfactory functional outcomes	Requirement for thin, pliable tissue to reduce fistula risk	Adequate reach to perineal and lower abdomen
Sidhoum et al., 2017 [11]	Patients requiring thin soft-tissue reconstruction	SCIP flap (free and pedicled)	Other local/perforator flaps	Perforator-based SCIP flap with variable paddle orientation	Reliable flap survival; low donor-site morbidity	Consistent perforator anatomy allows use in compromised beds	Good contour restoration with minimal bulk

Risk of Bias Assessment

Table 3 summarizes the methodological quality and risk of bias assessment of the included studies using the JBI Critical Appraisal Checklist for Case Series. Lichtenberg et al. conducted a technical case series and were rated as having a moderate risk of bias due to the small sample size and absence of a comparator group; however, surgical techniques and follow-up were clearly reported, providing valuable descriptive outcome data [9]. De Gelder et al. performed a retrospective single-center case series, also assigned a moderate risk of bias. The retrospective design introduced potential selection bias, but outcomes and complications were systematically documented [10]. Sidhoum et al. reported a retrospective anatomical and clinical case series with a moderate risk of bias, given the heterogeneous patient population and indications [11]. Despite limited comparative outcome data, the study provided detailed anatomical information that enhanced understanding of SCIP flap application in compromised soft-tissue beds. Collectively, the risk-of-bias assessment highlights that while all included studies were moderately limited by design and sample size, they provide consistent and clinically relevant insights into the use of pedicled and chimeric SCIP flaps for soft-tissue and fascial reconstruction.

**Table 3:** Risk of bias assessment JBI: Joanna Briggs Institute; SCIP: superficial circumflex iliac artery perforator Pedicled flap: flap transferred while maintaining its native vascular pedicle; Case series: observational study reporting outcomes of a series of patients without a control group

Study	Study Design	Risk of Bias Tool	Risk of Bias Rating	Justification
Lichtenberg et al., 2024 [9]	Technical case series	JBI Checklist for Case Series	Moderate risk of bias	Small sample size and lack of comparator; outcomes descriptive but surgical technique and follow-up clearly reported
De Gelder et al., 2024 [10]	Retrospective single-center case series	JBI Checklist for Case Series	Moderate risk of bias	Retrospective design with potential selection bias; outcomes and complications systematically reported
Sidhoum et al., 2017 [11]	Retrospective anatomical and clinical case series	JBI Checklist for Case Series	Moderate risk of bias	Heterogeneous population and indications; strong anatomical detail but limited comparative outcome data

Discussion

This systematic review demonstrates that pedicled and chimeric SCIP flaps are highly versatile and reliable for soft-tissue and fascial reconstruction in patients with vesicocutaneous bladder fistulas. Lichtenberg et al. reported successful closure of complex infra-umbilical and pelvic soft-tissue defects using a chimeric SCIP flap with a fascial component, highlighting stable soft-tissue coverage even in areas with chronic inflammation, fibrosis, and compromised vascularity [9]. Similarly, De Gelder et al. showed high flap survival and satisfactory functional outcomes in patients undergoing urogenital reconstruction, emphasizing the flap’s thin and pliable design that reduces the risk of fistula formation and allows adequate reach to the perineal and lower abdominal regions [10]. Sidhoum et al. demonstrated that SCIP flaps, whether free or pedicled, provide reliable survival and minimal donor-site morbidity, even in anatomically complex or compromised tissue beds [11]. Collectively, these studies suggest that the pedicled chimeric SCIP flap effectively addresses the pathophysiological challenges of vesicocutaneous fistulas, including impaired vascularity and scarred tissue, by providing both superficial coverage and structural reinforcement.

Compared with traditional reconstructive techniques, such as omental, rectus abdominis, or gracilis flaps, SCIP-based flaps offer distinct advantages. They provide thin, pliable tissue that contours well to pelvic anatomy, minimizing excessive bulk and preserving functional and aesthetic outcomes. Lichtenberg et al. highlighted that the fascial component enhances the mechanical support of bladder closure, potentially reducing recurrence rates [9], while De Gelder et al. reported that the flap effectively prevents urethral or bladder fistulas when applied using thin, pliable tissue suitable for urogenital reconstruction [10]. Moreover, Sidhoum et al. noted the consistency of SCIP perforator anatomy, which enables reliable flap harvest even in previously irradiated or surgically manipulated regions while maintaining low donor-site morbidity and avoiding sacrifice of major muscle groups [11]. These advantages collectively support the use of SCIP flaps as a refined alternative for reconstructing complex pelvic defects, particularly when traditional bulkier flaps may be less suitable [12].

Despite these encouraging outcomes, the current evidence has important limitations. All three studies were small, single-center case series with moderate risk of bias, limiting the strength and generalizability of conclusions. Lichtenberg et al. lacked a comparator group, making it difficult to determine relative efficacy against other reconstructive options [9]. De Gelder et al.’s retrospective design introduces potential selection bias and limits control over confounding variables [10]. Sidhoum et al. included heterogeneous patient populations, a wide range of indications, and varied flap configurations, complicating the interpretation of pooled outcomes [11]. Furthermore, key long-term functional endpoints such as urinary continence, sexual function, postoperative mobility, and quality of life were inconsistently reported. Complication profiles such as donor-site sensory changes, contour deformities, or delayed wound healing were also not uniformly assessed. These limitations highlight that the available evidence mainly offers technical feasibility insights rather than definitive comparative effectiveness.

Future research should focus on multicenter, prospective studies with standardized outcome measures and extended follow-up periods to better evaluate the durability of SCIP-based reconstructions. Comparative studies assessing pedicled and chimeric SCIP flaps against established options such as rectus abdominis, gracilis, or omental flaps would provide clearer guidance for flap selection in vesicocutaneous fistulas. Incorporation of patient-reported outcome measures, cost-utility analyses, and perfusion-based imaging (e.g., indocyanine green angiography) could help refine indications and optimize flap design. Developing collaborative databases or registry-based research platforms would be especially valuable given the relative rarity of vesicocutaneous bladder fistulas and the technical specificity of SCIP flap reconstruction. Integrating findings from Lichtenberg et al. [9], De Gelder et al. [10], and Sidhoum et al. [11] provides a strong foundation for improving surgical decision-making and underscores the potential of SCIP-based flaps as a reliable, anatomically appropriate, and functionally advantageous reconstructive option.

## Conclusions

Pedicled and chimeric SCIP flaps represent a reliable and versatile reconstructive option for vesicocutaneous bladder fistulas, particularly in patients with compromised tissue or prior pelvic interventions. These flaps provide both superficial coverage and fascial reinforcement, reduce donor-site morbidity, and allow tailored reconstruction in complex pelvic and infra-umbilical defects. Despite the limited number of high-quality studies, current evidence supports their use as a safe and effective alternative to traditional flaps. Further prospective research is needed to establish standardized surgical protocols, long-term functional outcomes, and comparative efficacy against other reconstructive techniques. Despite these constraints, preliminary evidence suggests that pedicled and chimeric SCIP flaps may represent a reliable and versatile option for complex vesicocutaneous bladder fistula repair. However, this conclusion is based on limited evidence from small case series
